# Supplementary material for: Mechanistic insight into carbon-carbon bond formation on cobalt under simulated Fischer-Tropsch synthesis conditions
Source: Nat Commun. 2020 Feb 6;11:750. doi: 10.1038/s41467-020-14613-5 (PMC7005166; doi:10.1038/s41467-020-14613-5)
Supplement: Supplementary file 1 — Supplementary Information [file 41467_2020_14613_MOESM1_ESM.pdf]

Supplementary information

Mechanistic insight into Carbon-Carbon bond formation on Cobalt under simulated Fischer-Tropsch Synthesis conditions

Weststrate et al.

Supplementary Note 1: XPS to identify the chemical nature of the carbon layer produced by heating of C_2H_x in the presence and absence of CO (g).

The C*1s* binding energy is sensitive to the chemical nature of nature of surface carbon. On Co(0001) we previously found that atomic(/carbidic) carbon causes a photoemission peak around 282.8 eV, whereas peaks due to graphene/graphite and disordered polymeric carbon typically appear around 284.4 eV [2]. Supplementary Figure 1 compares the C*1s* spectra obtained after heating the ethylene-covered surface in vacuum with that obtained after heating a C_2H_2 + $2H_{ad}$-covered surface in the presence of CO (g), to 630 K in both cases. The spectrum after heating in vacuum contains a single peak at 282.8 eV due to atomic carbon [1,2]. After heating in CO we instead find a mix of atomic and polymeric carbon. This shows that the C_4H_x surface species formed under the influence of CO tend to polymerize further during heating under hydrogen-lean conditions and produce ‘polymeric’ or even graphitic carbon upon heating to 630 K.

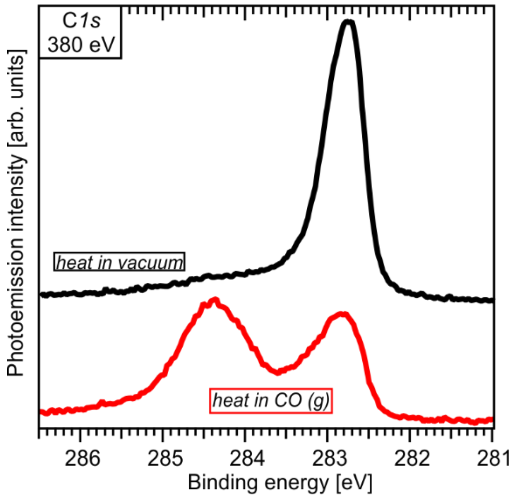

Supplementary Figure 1. Nature of carbon layer after heating to 630 K. C*1s* core level spectra after heating the C_2H_{xad}-covered surface in vacuum and in the presence of CO (g), respectively. The single peak at 282.8 eV found after heating in vacuum can be attributed to atomic carbon [2]. The additional peak at 284.4 eV is only found after heating in the presence of CO (g) and can be attributed to ‘polymeric’ or graphitic carbon [2].

Supplementary Note 2: Evaluation of the high resolution C*1s* spectra taken during heating of C_2H_x in CO (g)

Supplementary Figure 2 shows the as-measured high resolution spectra recorded at key temperatures during heating of the $C_2H_{2ad}/2H_{ad}$-covered surface in CO. The markers in Supplementary Figure 2 show individual datapoints with the fit of the data shown by a solid blue line. The Shirley background used to produce the background-subtracted spectra shown in Supplementary Figure 1(d) [main article] is shown as a grey line.

The spectrum after heating to 200 K shows that CO is adsorbed in both top (0.14 ML) and hollow (0.16ML) sites. In our previous detailed study of ethylene decomposition we reported that ethylidyne is formed as a minor side product of ethylene decomposition along with acetylene, but *only* when the

initial ethylene surface coverage is high. The small quantity of ethylidyne seen in the 200 K (with its two peaks marked in red in the upper panel) is due to this minor reaction path. The high resolution C*1s* spectrum of adsorbed acetylene on both Co(0001) on Ni(111) has been reported and discussed previously by several authors [1,3,4] and shown in isolation in the lower panel of Supplementary Figure 2. In line with these studies we attribute the high binding energy shoulders, located at +0.36 eV (and +0.7 eV) relative to the main peak to additional losses caused by excitation of the C-H vibration along with photoemission process [5].

Heating to 285 K in the presence of CO causes acetylene conversion to ethylidyne. A small quantity of unreacted can be seen in the spectrum, giving rise to small shoulder at the lower BE side of one of the ethylidyne-related peaks. Ethylidyne causes a distinct photoemission peak at a binding energy of 282.9 eV [shown in red the top central panel of Supplementary Figure 2], which is attributed to **C**(-CH_3), the carbon atom bound directly to the Co surface. As this carbon does not contain any hydrogens there are no high binding energy shoulders due to excitation of the C-H vibration. Instead, other authors have observed a small shoulder at +0.15 eV for a pure ethylidyne layer on Rh(111) with an intensity that is 13% of the main peak [6], attributed to excitation of the C-C vibration of ethylidyne (0.15 eV = 1210 cm^{-1}). We cannot resolve this in our experiments because of the presence of the residual acetylene which causes a peak around the same binding energy. We accounted for this by adding a peak at +0.15 eV and with a signal intensity that is 13% of the main peak. The peak at 283.5 eV (blue in Supplementary Figure 2, top-centre) shows the vibrational finger print of a CH_3 group, with shoulders at +0.39 and +0.8 eV, respectively, due to excitation (double excitation) of its C-H vibrations [1,6,7]. Co-adsorbed CO causes a significant downward shift of the binding energy, as reported before [6] for ethylidyne on Rh(111) with CO co-adsorbed, also discussed in more detail in the next section.

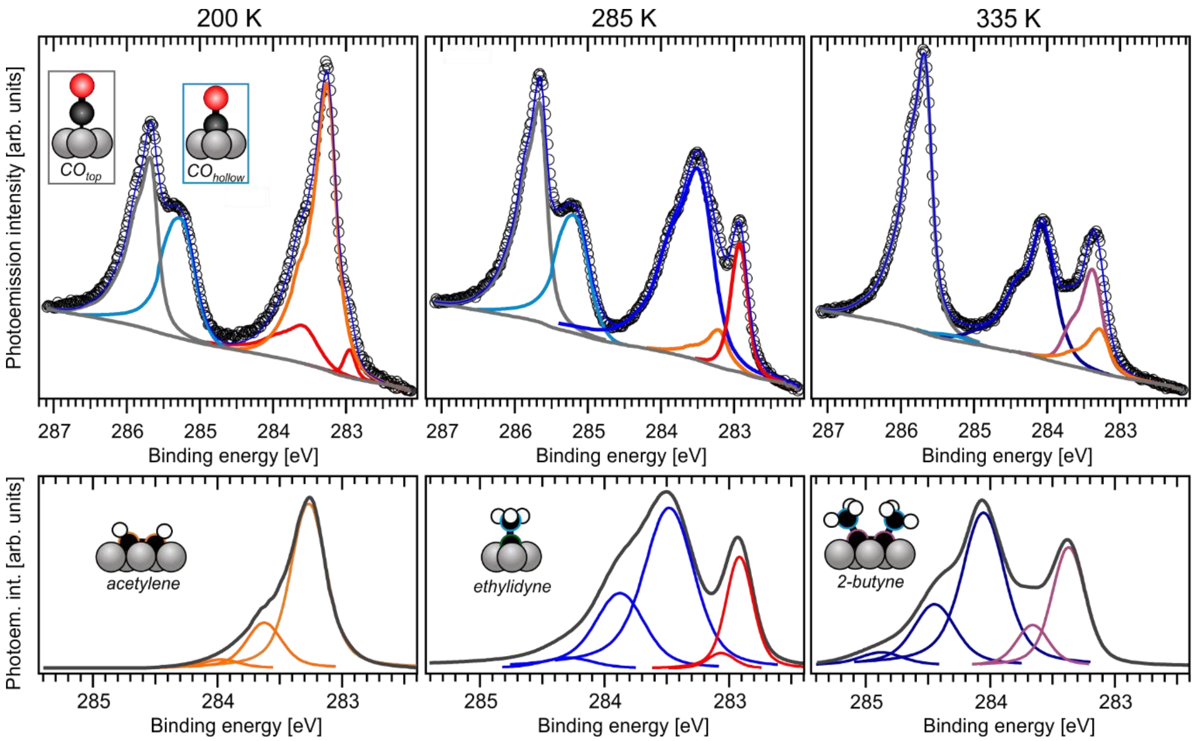

Supplementary Figure 2. Deconvolution of high resolution C*1s* spectra. Deconvolution of the high resolution XPS spectra (hν = 325 eV) obtained after heating the C_2H_{2ad} + $2H_{ad}$ layer to the indicated temperatures in the presence of 1×10^{-7} mbar CO. The CO pressure was removed after reaching the desired temperature. After cooling in vacuum, the spectra were recorded at 90 K to minimize thermal broadening.

The spectrum recorded after heating to 335 K is attributed to 2-butyne, produced by coupling of two ethylidyne species. The difference in the spectrum attributed to ethylidyne and 2-butyne twofold: (i) the peak due to **C**-CH_3 of ethylidyne, at 282.9 eV, disappears and a new peak appears at 283.4 eV purple in Supplementary Figure 2, right), close to the binding energy value of the central carbon atom in adsorbed propyne (H_3C-**C**-CH) (see next section and ref. [1]). This is to be expected, since chemically speaking the central carbon atoms in adsorbed propyne and adsorbed 2-butyne are very similar. (ii) The shape of the methyl peak around 284.1 eV indicates that the methyl group has remained intact. The shift of its binding energy, back to a value that is commonly found for methyl substituents, is caused by the lower θ_{CO} at 335 K. The absence of CO_{hollow} seems to be particularly important, as discussed hereafter. Although both spectra were recorded with the same resolution settings the shape of the spectrum of the methyl group is better resolved for 2-butyne than it is for ethylidyne. This is attributed to a slight broadening of these peaks due to co-adsorbed CO, as also found when CO is co-adsorbed alongside propyne, as discussed in the next section.

We previously concluded that hydrocarbons on Ni(111) react in a similar manner as they do on cobalt, and in XPS the binding energies found for the same adsorbate are often practically identical (compare, e.g. ethylene on Ni(111) and Co(0001), [4] vs [1]). This makes the high resolution C*1s* spectra of both acetylene and 2-butyne adsorbed on Ni(111) reported by the Hirschmugl et al.[8] highly relevant to validate our assignment. These authors did not use the fermi edge to calibrate the binding energy scale, but since they also report the spectrum for acetylene we can use the positions they report for the 2-butyne-related peaks on Ni(111) *relative* to those of acetylene to compare with our data. By re-calibrating the literature reference in this way we find a value of 283.4 eV for the two central carbon atoms of 2-butyne adsorbed on Ni(111), identical to the value we find on Co(0001). These authors report the presence of a shoulder to the main peak of the central carbon atoms, at 283.6 eV, the same shoulder that we find in our spectrum attributed to 2-butyne. Their explore the origin of this peak at +0.2 eV from the main peak in more detail, and conclude that "the lines from the central C atoms in Ni(111)-2-butyne could not be assigned to a dominating single vibrational mode. Instead a ionization process is proposed which involves the simultaneous excitation of two vibrational modes of the core ionized molecule."[8]. The two methyl groups of 2-butyne on Ni(111) appear at 284.2 eV. On Co(0001) we find a value of 284.1 eV, where we attribute the 0.1 eV lower value relative to Ni(111) to the presence of CO_{top} in our experiments, which, as discussed hereafter, causes a minor downward shift of the binding energy of methyl substituents. Thus, the XPS spectra reported for 2-butyne on Ni(111) support the identification of 2-butyne as the product of CO-induced coupling of two ethylidyne adsorbates in our experiments on Co(0001).

Supplementary Note 3: The influence of CO_{ad} on the C*1s* binding energy of methyl substituents

In their study of the influence of CO_{ad} on the C*1s* photoemission spectrum of ethylidyne adsorbed on Rh(111) [6] Wiklund et al. report that, in particular, the signal due to the methyl group shifts to lower binding energies when CO is co-adsorbed alongside ethylidyne. We attribute the downward shift of the methyl binding energy in ethylidyne to the same phenomenon. To further corroborate this point we studied how the binding energy of the methyl group of adsorbed propyne shifts as a function of CO coverage. The propyne layer was prepared by heating a surface covered with 0.08 ML propylene to 220 K, a procedure which results in a surface coverage of 0.08 ML propyne + 0.16 ML H_{ad} [1]. Supplementary Figure 3(a) shows a top view of the C*1s* spectra that were obtained during a subsequent CO dose at 100 K. It can be seen that CO, in the low coverage regime where only top-CO is present, causes a slight, ~0.1 eV downward shift of the methyl group binding energy. Hollow sites become populated when the CO coverage increases, and this coincides with an additional ~0.4 eV

downward shift of the binding energy of the methyl group of adsorbed propyne adding up to a total downward shift of 0.5 eV. Supplementary Figure 3(b) shows some typical spectra (spectrum number indicated in the graph) to better assess how the shape of the C*1s* spectrum of propyne changes due to the presence of co-adsorbed CO. Apart from the shift of the peak position we found a slight overall broadening of the spectra which is assigned to the influence of CO. A similar slight broadening can be observed when comparing the high resolution spectrum of acetylene before [300 K spectrum, Supplementary Figure 1(a) main article] and after CO dosing [200 K spectrum, Supplementary Figure 1(d) main article].

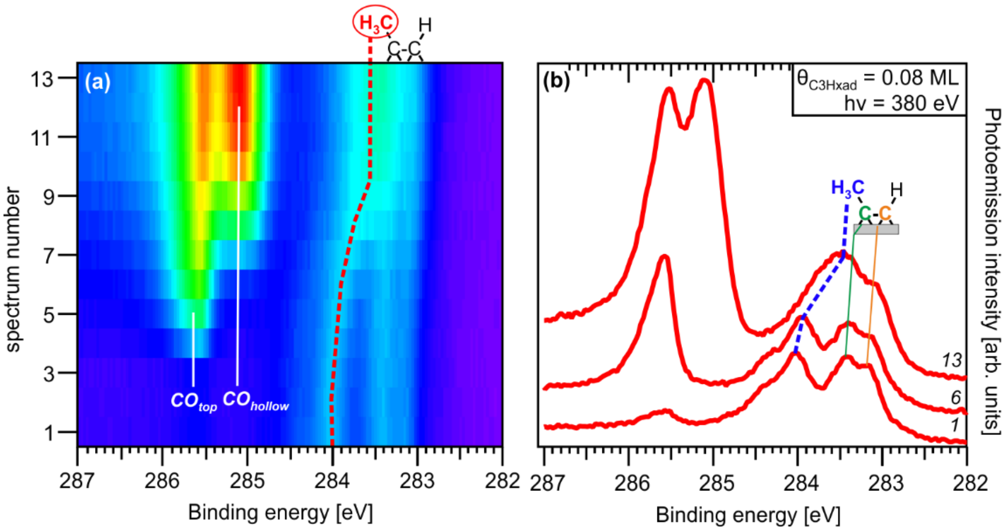

Supplementary Figure 3. The influence of CO_{ad} on the binding energy of methyl groups. (a) Top view of the C*1s* region during dosing of CO onto a 0.08 ML propyne-covered surface (hv = 380 eV, T=88 K). The red line indicates the position of the photoemission signal due to the methyl substituent of propyne. (b) Individual spectra taken from the series shown in (a) show details of how the shape of the spectrum changes with CO coverage.

Supplementary Note 4: TPRS and RAIRS using 2-butyne

Our spectroscopic observations suggest that 2-butyne is produced when a C_2H_{2ad}/2 H_{ad} layer is heated in the presence of CO. We performed several experiments using this 2-butyne directly (liquid, boiling point 27°C) to further corroborate the formation that 2-butyne forms as the product of ethylidyne dimerization. A series of TPR spectra recorded for different doses of 2-butyne revealed that the saturation coverage of chemisorbed 2-butyne is 0.15 ML. With an initial C_2H_x concentration of 0.12 ML the upper limit to the concentration of the 2-butyne product in our experiments using CO is 0.06 ML. We therefore performed our reference experiments using a 2-butyne coverage of ~0.05 ML to mimic the C_2H_x in CO-experiment as closely as possible. In addition, since CO is present in the C_2H_x experiment we also studied how the presence of CO affects the TPRS and IR absorption spectrum of adsorbed 2-butyne.

Supplementary Figure 4(a) compares the H_2 desorption trace obtained during heating of a saturated C_2H_{xad} surface in CO (g) with that obtained during heating of a 0.05 ML 2-butyne-covered surface both in vacuum and in the presence of CO (g). The first H_2 desorption peak at 350 K found during heating in vacuum is attributed to (partial) dehydrogenation of the methyl substituents, in analogy to a very similar peak found for methyl dehydrogenation of adsorbed propyne, see refs. [1,9]. This peak shifts to 365 K when 1×10^{-7} mbar CO is present the exact same temperature as found when the C_2H_{xad} layer is

heated in the same CO pressure. Further dehydrogenation of 2-butyne occurs gradually between 400-620 K. The similarity of the H_2 desorption spectra of C_2H_{xad} in CO and 2-butyne in CO supports the notion that 2-butyne is produced from C_2H_{xad} in the presence of CO.

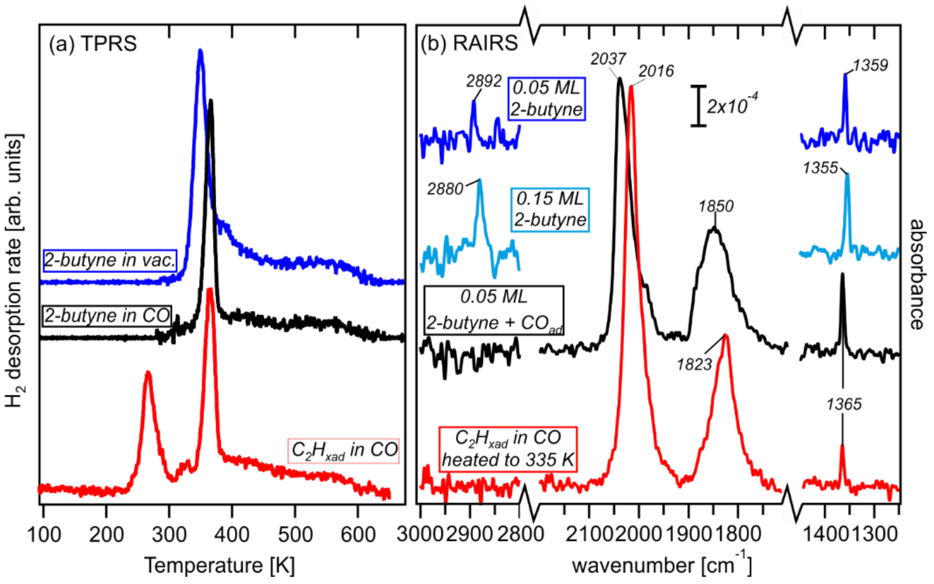

Supplementary Figure 4. Reference experiments using 2-butyne. (a) Comparison of the H_2 desorption spectrum obtained during heating of 0.12 ML C_2H_2 + 0.24 ML H_{ad} in the presence of CO (g) with those obtained during heating of 0.05 ML 2-butyne in vacuum and in CO, respectively. (b) A comparison of the IR absorption spectrum of C_2H_{xad} after heating to 335 K and cooling in CO (g) with that of 0.05 ML 2-butyne + CO_{ad}, 0.05 ML 2-butyne and 0.15 ML (sat.) 2-butyne, respectively (heating rate = 0.2 $K \cdot s^{-1}$, $p_{CO} = 1 \times 10^{-7}$ mbar).

Supplementary Figure 4(b) compares the IR absorption spectrum of C_2H_{xad} after heating in CO to 335 K with spectra obtained after adsorbing 2-butyne. The saturated 2-butyne spectrum (0.15 ML) shows a band at 2880 cm^{-1} due to the symmetric C-H stretch of $-CH_3$. Along with this, a clear band is found at 1355 cm^{-1}, attributed to the symmetric C-H bending (umbrella) mode of $-CH_3$. For 0.05 ML 2-butyne this band decreases somewhat in intensity and is shifted to 1359 cm^{-1}. The 2880 cm^{-1} band shifts to 2892 cm^{-1} and becomes much weaker. Co-adsorption of CO along with 0.05 ML 2-butyne causes the stretching mode to disappear, an effect of CO that has been reported previously [10,11]. The bending mode shifts to 1365 cm^{-1}, the exact same frequency as found after heating C_2H_{xad} to 335 K and cooling in CO. The CO-related bands between 2100-1750 cm^{-1} shows occupation of both top (2016-2037 cm^{-1}) and threefold hollow (1823-1850 cm^{-1}) sites in both cases. We take the close similarity of both the H_2 desorption due to 2-butyne decomposition and the similarity of the IR absorption spectra as further evidence that supports the conclusion that CO-induced reactions of C_2H_{xad} species produce 2-butyne as a reaction product.

Supplementary Note 5: Quantitative analysis of XPS, photoelectron diffraction + discussion of the ordered layer associated with the c(4×2)diffraction pattern

The TP-XPS data shown in Figure 1(d) [main article] can be used to determine the coverage all adsorbates except hydrogen. These were derived from the TPRS data, as discussed hereafter. Such highly detailed information allows us to determine the kinetics of both ethylidyne formation and its dimerization. It furthermore helps to understand the meaning of the ordered LEED pattern found on

the surface. A number of issues have to be taken into account when quantifying our data and these are discussed in detail here.

The TP-XPS data was recorded using 380 eV, whereas 325 eV was used for high resolution spectra. In general, the use of such low photon energies among other things the advantage of a ~40 times higher photoemission cross section for C*1s* compared to a conventional Al Kα source (hv=1486.6 eV), and the resulting low kinetic energy of the photoelectrons produced (95 eV and 40 eV) also increases surface sensitivity. The drawback of the low kinetic energy is that photoelectron diffraction effects are particularly strong, a complication to quantitative analysis. We previously verified that the C*1s* signal intensity of CO is directly proportional to the CO surface coverage so we can exclude that photoelectron diffraction affects quantification of the CO-related peaks based on their peak area [12]. This notion is confirmed by the fact that quantitative analysis of the O*1s* spectra (Supplementary Figure 8) gives the exact same coverage values as those obtained from analysis of the C*1s* spectra.

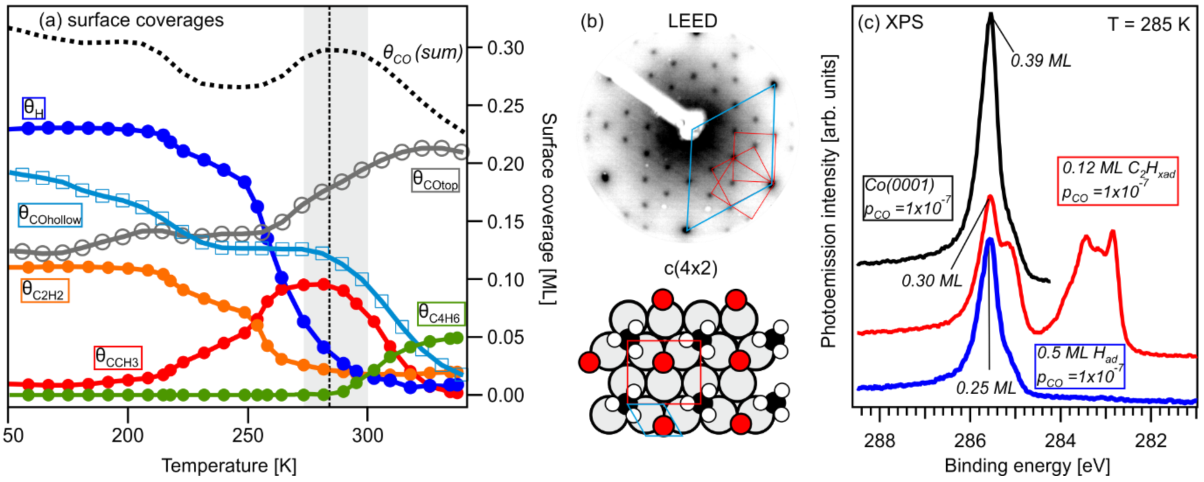

Supplementary Figure 5. Analysis of adsorbate concentration and ordering. (a) Concentration of all adsorbates during heating of C_2H_{xad} in CO. (b) c(4×2) LEED pattern (80 eV) and corresponding structure of the mixed CO/CCH_3 adlayer visible between 270-305 K during heating of C_2H_{xad} in CO. (c) Comparison of the C*1s* spectrum at 285 K during heating of a C_2H_{xad} layer in CO with the clean and 0.5 ML H_{ad}-covered surface heated in CO, respectively. (p_{CO} = 1×10^{-7} mbar, heating rate = 0.2 $K\cdot s^{-1}$)

For C_xH_y adsorbates the effect of photoelectron diffraction is often very strong. A good example of how photoelectron effects influence signal intensities can be seen in Supplementary Figure 2 (main article) and Supplementary Figure 2: the peak areas for the two carbon atoms of adsorbed ethylidyne are clearly different, despite the fact that their concentrations are the same. In addition to this, their intensity ratio is a strong function of the exact photon energy used (= wavelength of the photoelectrons), as shown in refs. [6,13]. This implies that we cannot use the signal intensity directly to determine the concentration of the different C_xH_{yad} species formed during heating. We therefore adopted a different approach: we first obtain the spectral shape of each of the species present of the surface. A linear combination of the different peak shapes is then used to deconvolute the spectra in the transient regimes where different species co-exist.

It is easy to obtain the spectrum of a pure acetylene layer: on an *ethylene-saturated* surface decomposition at 180 K initially produces a product mix consisting of 96% acetylene and ~4%

ethylidyne [1], but heating to 300 K causes ethylidyne to decompose into a pure acetylene layer [see also Supplementary Figure 7 and Supplementary Figure 1(a)]. After co-adsorption of CO, which causes slight changes to the spectral shape of adsorbed acetylene, we can then obtain a reference spectrum of acetylene in the presence of CO. Alternatively, we can also dose less ethylene. When the initial ethylene concentration is ~0.06 ML decomposition around 180 K yields a pure acetylene (+2H_{ad}) layer[1]. After co-adsorption of CO the reference spectrum can then be obtained. Ethylidyne was never present alone in our experiments, but the XPS experiments show that its concentration reaches a maximum after heating to 285 K in the presence of CO. The ethylidyne spectrum was obtained subtracting the acetylene reference spectrum multiplied with an appropriate scaling factor so that the shape of the residual corresponds to that of ethylidyne [6]. The spectra between 200-300 K where both species co-exist were then analysed by using a linear combination of the spectra of the two pure species. Due to the influence of CO_{ad} on the binding energy of the methyl group, discussed hereafter, it was necessary to shift the position of the methyl signal of ethylidyne. The reference spectrum for 2-butyne was obtained by subtracting the spectrum at 335 K with the same quantity of acetylene, and the result was used in a linear combination with the spectrum of ethylidyne to evaluate the spectra between 290-350 K.

This approach provides us with relative concentrations of the adsorbates. But since the mass spectrometer shows only desorption of H_2 we can exclude that carbon-containing species (other than CO) leave the surface. We can therefore safely assume that the sum of all C_2H_{xad} species remains constant at the initial value of 0.12 ML. The absolute concentration of the C_xH_y adsorbates can thus be determined by using the mass balance given by eq. S1:

$$\theta_{C2H2} + \theta_{C2H3} + 2\cdot\theta_{C4H3} = 0.12 \qquad \text{(eq. 1)}$$

Adsorbed hydrogen atoms cannot be directly detected by XPS. Instead, we can use a similar mass balance to determine θ_H, eq. S2:

$$\theta_H = 0.24 - \theta_{C2H3} - \text{desorbed } H_2 \qquad \text{(eq. 2)}$$

The results of this quantitative analysis of adsorbate concentrations is shown in Figure 6 (main article), Supplementary Figure 5(a) and in Supplementary Figure 6.

We find that a c(4×2)) LEED pattern during our experiments in which ~0.12 ML ethylidyne is formed in the presence on CO. The same pattern can be found on Rh(111). Here ethylidyne is the only product of ethylene decomposition after heating to 230 K. It forms an ordered overlayer structure with a coverage of 0.25 ML and a (2×2) pattern LEED[6,14]. Co-adsorption of CO alongside 0.25 ML ethylidyne on Rh(111) leads to the formation of a c(4×2) pattern in LEED. The CO coverage in this structure is 0.25 ML, and IV-LEED, electron energy loss spectroscopy (EELS) and XPS all confirm that CO exclusively resides in the threefold hollow sites [6,14].

In our experiments the situation is less straightforward. The c(4×2) unit cell contains 4 cobalt surface atoms which implies that the local concentration of ethylidyne and CO_{ad} in this structure is 0.25 ML. But since the maximum CCH_3 concentration that can be reached in our experiments is limited by the initial concentration of acetylene, 0.12 ML[1], we conclude that the LEED pattern between 270-305 K is caused by *islands* of co-adsorbed CO_{ad} and ethylidyne with a local concentration of 0.25 ML of both. This leaves the other ~50% of the surface be covered by a disordered adsorbate layer which contains CO_{ad}, H_{ad} and C_2H_{xad} with concentrations that change as a function of temperature.

The XPS data shed more light on this issue. Supplementary Figure 5(b) compares the C*1s* spectrum of C_2H_{xad} heated in 1×10^{-7} mbar CO to 285 K with that of the clean Co surface and a 0.5 ML H_{ad}-covered

surface heated to 285 K in 1×10^{-7} mbar CO. We can see that at 285 K CO occupies predominantly the top sites (285.5 eV [12]) in the absence of $C_xH_{yad.}$ Instead, for the C_2H_{xad} experiment we find a prominent peak at 285.1 eV after heating to 285 K, due to CO adsorbed in hollow sites [12]. Since this peak is only seen at 285 K when C_2H_{xad} is present it must be correlated with the presence of CCH_{3ad}, i.e. CO resides in hollow sites in the CO/CCH_3 co-adsorption structure that causes a c(4×2) pattern in LEED, and the structure of this structure must be similar to that reported for (111), shown in fig S5(a).

Supplementary Note 6: Description of the mean field microkinetic model used to determine reaction barriers for 2-butyne hydrogenation and (CO-induced) acetylene hydrogenation and ethylidyne coupling.

A simple microkinetic mean field model was constructed to estimate reaction barriers, based on the assumption that the reactions we observe are irreversible. It should be noted that both 2-butyne hydrogenation and acetylene hydrogenation to ethylidyne require a sequence of elementary reaction steps that involve the breaking and making of multiple C-H bonds. Since we simulate both processes with a single step the barrier found for these steps are therefore representative of the slowest step in the sequence.

2-butyne hydrogenation

For 2-butyne hydrogenation (Figure 5, main article) we assumed a simple differential equation:

$d\theta_{C4H6}/dt = k_{butyne}\cdot \theta_{C4H6}$

where we assumed that θ_H is high and constant due to the presence of a 1×10^{-1} mbar H_2 atmosphere.

The model result shown in Supplementary Figure 5 (main article) was obtained with a barrier of 102 $kJ\cdot mol^{-1}$ in combination with $\nu = 1\times10^{11}\ s^{-1}$.

Acetylene → ethylidyne → 2-butyne

The conversion of acetylene to ethylidyne followed by ethylidyne dimerization (Figure 6, main article) was described using the set of differential equations given below:

$d\theta_H/dt = \theta_H(0) - k_{ethform}\cdot\theta_H\cdot \theta_{c2H2} - k_{Hdes}\cdot\theta^2_H$

$d\theta_{C2H2}/dt = \theta_{C2H2}(0) - k_{ethform}\cdot\theta_H\cdot \theta_{C2H2}$

$d\theta_{C2H3}/dt = \theta_{C2H3}(0) + k_{ethform}\cdot\theta_H\cdot \theta_{C2H2} - 2\cdot k_{couple}\cdot \theta^2_{C2H3}$

$d\theta_{C4H6}/dt = k_{couple}\cdot \theta^2_{C2H3}$

where:

$k_n = \nu_n \cdot\exp(-E_{act}(n)/RT)$

and:

$T = T_0 + \beta\cdot t$

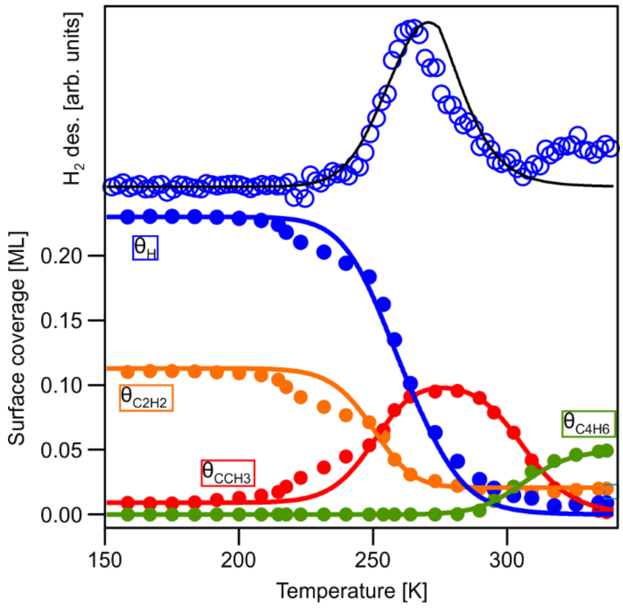

Supplementary Figure 6. Microkinetic modelling of adsorbate concentrations. A comparison of the measured (datapoints) and simulated (solid lines) concentrations of reactants and products during heating of an acetylene + 2 H_{ad}-covered surface in 1×10^{-7} mbar CO (0.2 $K\cdot s^{-1}$).

Definition of the variables:

T = temperature [K]

t = time [s]

β = heating rate [$K\cdot s^{-1}$] (0.2 here)

ν_n = pre-factor for reaction n [s^{-1}]

$E_{act}(n)$= activation energy for reaction n [$J\cdot mol^{-1}$]

R = molar gas constant [8.314 $J\cdot K^{-1}\cdot mol^{-1}$]

Supplementary Table 1. *Pre-factor and activation barrier combinations obtained from the kinetic model.*

Reaction acronym	reaction	ν [s^{-1}] optimized	E_{act} [$kJ\cdot mol^{-1}$] optimized	ν [s^{-1}]	E_{act} [$kJ\cdot mol^{-1}$]
butyne	butyne + $2H_{ad}$ → butene	**1×10^{11}**	102	1×10^{13}	118
Hdes	2 H_{ad} → H_2 (g)	1×10^{13}	73	1×10^{13}	73
ethform	C_2H_{2ad} + H_{ad} → CCH_{3ad}	**1×10^{10}**	**53**	1×10^{13}	66
couple	2 CCH_{3ad} → H_3C-CC-CH_3	**1×10^{13}**	**82**	1×10^{10}	65

In Supplementary Figure 6 we compare the concentrations of the reactants as derived from the experiment with the output of this model with optimized pre-factor activation energy combinations. Supplementary Table 1 provides some more information about the values of pre-factor and activation

energy. The error in our estimated barriers was evaluated by varying the assumed pre-factor followed by optimization of the activation energy value. Some of the thus obtained values are provided in Table 1 as well. The kinetic parameters for H_2 desorption were derived from a reference experiment where 0.5 ML Had was heated in the presence of 1×10^{-7} mbar CO (see Figure 1(c) in main article and ref. 15). The hydrogenation of acetylene does not go to 100%, presumably because H_2 desorption depletes the surface hydrogen concentration before all acetylene has reacted. This complexity is difficult to capture in our simple model. Instead, for our simple approach we use the part of acetylene that is converted as the initial acetylene concentration. The θ_{C2H2} shown in Figure 6 (main article), Supplementary Figure 5 and Supplementary Figure 6 was obtained by adding the unreacted fraction of acetylene to the output of the kinetic model.

Supplementary Note 7: Beam-induced ethylene → ethylidyne + H_{ad}: CCH_3 reactivity in the absence of CO_{ad}.

In our previous study of ethylene adsorption and decomposition on Co(0001) we found that ethylidyne forms as a minor (4%) side product of ethylene decomposition[1], but only when the initial ethylene concentration is high. In that study we made sure that the results were not influenced by beam damage.

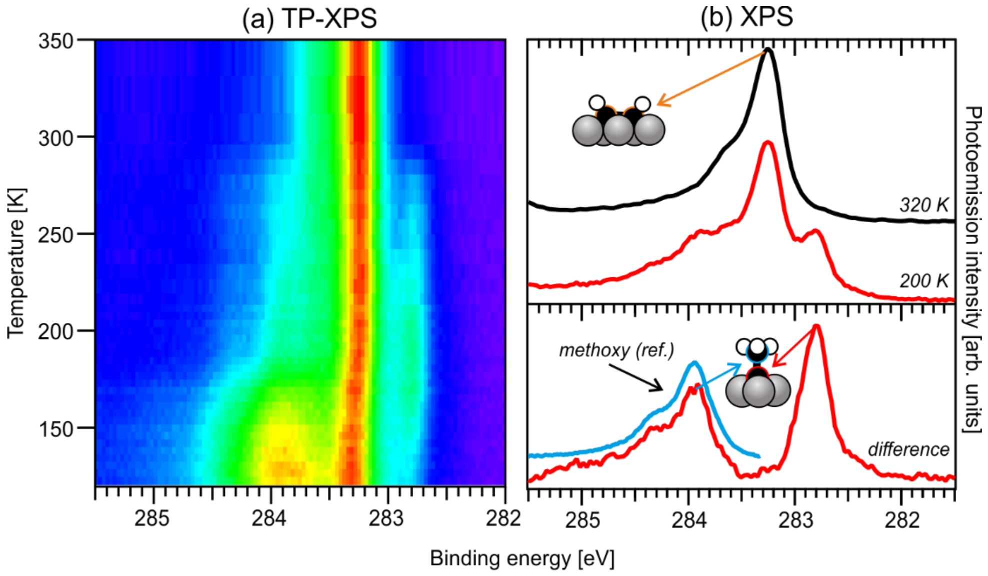

Supplementary Figure 7. Reactivity of ethylidyne in absence of CO (a) Top view of the C*1s* region during slow heating (0.1 K·s^{-1}) of an ethylene-covered surface. Beam-induced ethylene decomposition produces ethylidyne with two characteristic peaks at 282.8 and 283.9 eV, respectively. (b) individual XP spectra at 200 K (acetylene + ethylidyne) and 320 K (acetylene only). The difference spectrum shown in the lower panel corresponds to the spectrum of ethylidyne. The high resolution C*1s* spectrum of adsorbed methoxy (O-CH_3 [1,16]) is added to show the characteristic shape of the C*1s* spectrum due to a methyl substituent (hv = 380 eV).

In a reference experiment we instead used the fact that ethylene is slowly converted to ethylidyne during prolonged irradiation with the intense photon beam (hv=380 eV) to create a Co(0001) surface that is covered with a significant ethylidyne concentration but in the absence of CO_{ad}. We found that only adsorbed ethylene is sensitive to beam damage (a process which produces ethylidyne + H_{ad}) while

acetylene, ethylidyne and 2-butyne are unaffected by the beam. Supplementary Figure 7(a) shows a top view of the C*1s* spectra obtained during very slow heating of an ethylene-saturated surface while measuring the C*1s* spectral region using a photon energy of 380 eV, in this way maximizing the exposure to the x-ray beam to maximize the beam-induced ethylene decomposition, a reaction that produces ethylidyne + H_{ad}. Since CO_{ad} is absent in this experiment we can use these results to find out how ethylidyne reacts in the absence of CO. The TP-XPS shows that ethylidyne-related peaks disappear around below 280 K. This coincides with the increase of the acetylene peak, and the spectrum at 320 K [Supplementary Figure 7(b)] indeed corresponds to a pure acetylene layer. So instead of coupling to produce 2-butyne we find that ethylidyne dehydrogenates to acetylene (more stable than ethylidyne when CO is not there) below the temperature needed for ethylidyne dimerization. This corroborates the conclusion that CO stabilizes ethylidyne. This (i) causes it to form in the first place, and (ii) keeps it intact up to 310 K in our TP-experiment so that dimerization can occur to produce 2-butyne.

Supplementary Note 8: CO_{ad}, spectator or reactant?

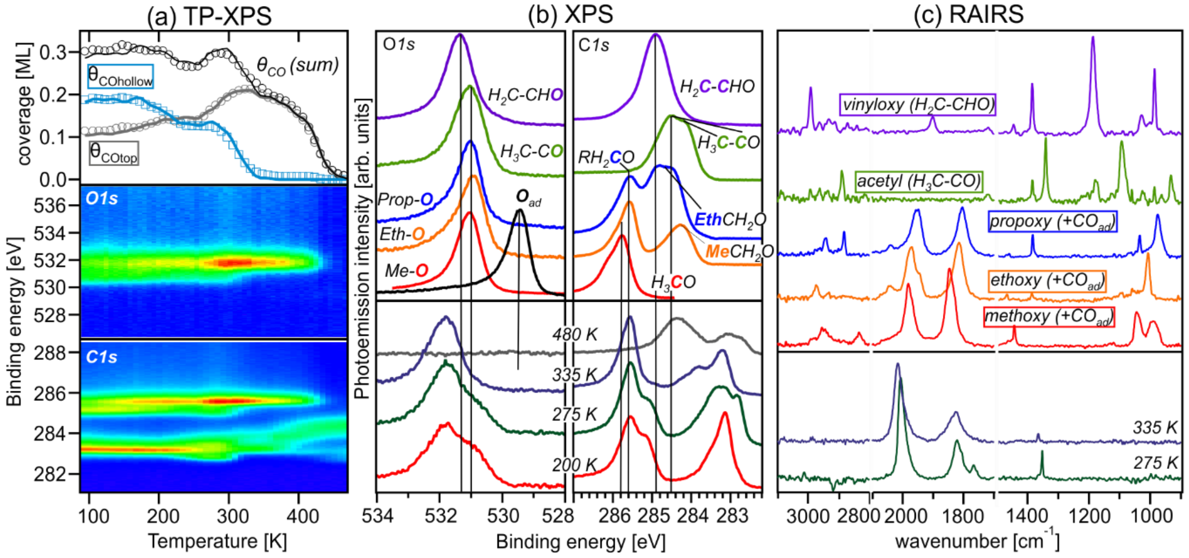

Supplementary Figure 8. Experimental information on oxygenate formation. (a) Top view of O*1s* (hv = 650 eV) and C*1s* (hv=380 eV) spectra during heating of $C_2H_{2ad}/2H_{ad}$ in CO. The top panel shows a comparison of θ_{COtop} and $\theta_{COhollow}$ derived from the C*1s* (markers) and O*1s* (solid line) respectively. Panel (b) shows a comparison of the O*1s* and C*1s* spectra at key temperatures spectra of O_{ad}, methoxy, ethoxy, propoxy, acetyl and vinyloxy, respectively. Panel (c) compares the IR absorption spectra during heating of $C_2H_{2ad}/2H_{ad}$ in CO with the same reference compounds, where CO was co-adsorbed alongside the alkoxy adsorbates to mimic the experimental conditions during the $C2H_{xad}$/CO experiment as closely as possible.

The experimental data exclude the possibility of CO reacting with C_2H_{xad} to form (C3) oxygenate species. This would imply the formation of oxygen-containing intermediates, and eventually C-O bond scission [7] to produce atomic oxygen on the surface. The investigate this in more detail we have to consider the information provided by the O*1s* spectra that were also recorded during slow heating of $C_2H_{xad}/2H_{ad}$ in CO. Supplementary Figure 8(a) shows a top view of the O*1s* region together with the C*1s* core level region during heating. The O*1s* signals can be exclusively attributed to CO, adsorbed in top (532.0 eV) and hollow (530.9 eV) [12] sites, respectively. We find an excellent agreement between the top/hollow concentrations obtained from the C*1s* [markers in the top panel of Supplementary Figure 8(a)] and the O*1s* [solid line, Supplementary Figure 8(a)], confirming that they are due to the

same species. In Supplementary Figure 8(b) we compare the O*1s* and C*1s* spectra at key points during heating of a $C_2H_2/2H_{ad}$-covered Co(0001) surface in CO (lower half) with spectra obtained for several oxygen-containing adsorbates, namely atomic oxygen, methoxy, ethoxy and butoxy, as well as acetyl and vinyloxy (upper half), the latter two being surface intermediates that form during acetaldehyde (H3C-CH=O) decomposition on Co(0001) [7]. With the O*1s* signal due to atomic oxygen at 529.4 eV [23], it is clear that atomic oxygen is not formed at any stage of the experiment. The O*1s* binding energy of the oxygenates overlaps with the signal due to CO adsorbed in hollow sites, so their formation cannot be excluded based on the O*1s* spectra alone. However, the carbon atom that is directly attached to the oxygen atom in the alkoxy adsorbates is located around 285.6-285.7 eV, the region where CO on top sites is found in the C*1s* spectrum. This means that the formation of alkoxy adsorbates would cause an increase of the signal in the CO_{hollow} region in O*1s* but in the C*1s* it would increase the signal in the CO_{top} region, resulting in deviation between the C*1s* and O*1s*-derived top/hollow concentrations. Since this is not seen in the top panel of Supplementary Figure 8(a) we can exclude that alkoxy intermediates form. The C*1s* signals of the oxygenate intermediates formed via acetaldehyde decomposition appear at binding energies in between those of CO_{hollow} and C_xH_{yad}, so formation of such intermediates would be directly visible in the C*1s* spectra.

The final piece of evidence that speaks against CO incorporation comes from the IR measurements shown in Supplementary Figure 8(c). Oxygen-containing species, (with CO co-adsorbed to better mimic the situation during heating of $C_2H_{2ad}/2H_{ad}$ in CO), always show strong characteristic bands in the region between 1200-1000 cm^{-1} due to C-O and C-C stretching modes[24]. No absorption bands were seen in this region during heating of $C_2H_{2ad}/2H_{ad}$ in CO, providing further support for the notion that (endothermic[25]) CO insertion does not play a role in the experiments presented here.

Supplementary References

1. Weststrate, C. J., Ciobîcă, I. M., van de Loosdrecht, J. & Niemantsverdriet, J. W. Adsorption and Decomposition of Ethene and Propene on Co(0001): The Surface Chemistry of Fischer–Tropsch Chain Growth Intermediates. *J. Phys. Chem. C* **120,** 29210–29224 (2016).
2. Weststrate, C. J. *et al.* Atomic and polymeric carbon on Co(0001): Surface reconstruction, graphene formation, and catalyst poisoning. *J. Phys. Chem. C* **116,** 11575–11583 (2012).
3. Borg, A. *et al.* Acetylene chemisorption and decomposition on the Co(11-20) single crystal surface. *Surf. Sci.* **499,** 183–192 (2002).
4. Lorenz, M. P. *et al.* Ethene adsorption and dehydrogenation on clean and oxygen precovered Ni(111) studied by high resolution x-ray photoelectron spectroscopy. *J. Chem. Phys.* **133,** 11–16 (2010).
5. Denecke, R. Surface chemistry studied by in situ X-ray photoelectron spectroscopy. *Appl. Phys. A* **80,** 977–986 (2005).
6. Wiklund, M., Beutler, A., Nyholm, R. & Andersen, J. N. Vibrational analysis of the C *1s* photoemission spectra from pure ethylidyne and ethylidyne coadsorbed with carbon monoxide on Rh(111). *Surf. Sci.* **461,** 107–117 (2000).
7. Weststrate, C. J. *et al.* Ethanol decomposition on Co(0001): C-O bond scission on a close-packed cobalt surface. *J. Phys. Chem. Lett.* **1,** 1767–1770 (2010).
8. Hirschmugl, C. J., Paolucci, G., Esch, F., Lizzit, S. & Schindler, K.-M. Vibrational fine structure on C_{1s} core-level photoemission : Ni(111)-ethyne and Ni(111)-butyne. *Surf. Sci.* **488,** 43–51 (2001).

9. Weststrate, C. J. & Niemantsverdriet, J. W. CO as a Promoting Spectator Species of C_xH_y Conversions Relevant for Fischer-Tropsch Chain Growth on Cobalt: Evidence from Temperature-Programmed Reaction and Reflection Absorption Infrared Spectroscopy. *ACS Catal.* **8,** 10826–10835 (2018).

10. McCoustra, M. R. S., Ainsworth, M. K., De La Cruz, C., Chesters, M. A. & Sheppard, N. An infrared study of ethene and CO coadsorption on Pt(111) and a Pt/SiO_2 catalyst: ambiguities in the interpretation of difference spectra. *Surf. Sci.* **437,** 9–17 (2002).

11. Mims, C. A., Weisel, M. D., Hoffmann, F. M., Sinfelt, J. H. & White, J. M. Site Blocking Effects in Ethylidyne Decomposition Kinetics on Ru(001):In-Situ Study with Infrared Reflection Absorption Spectroscopy at Elevated Pressure. *J. Phys. Chem* **97,** 12656–12659 (1993).

12. Weststrate, C. J., van de Loosdrecht, J. & Niemantsverdriet, J. W. Spectroscopic insights into cobalt-catalyzed Fischer-Tropsch synthesis: A review of the carbon monoxide interaction with single crystalline surfaces of cobalt. *J. Catal.* **342,** 1–16 (2016).

13. Andersen, J. N. *et al.* Vibrational fine structure in the C 1s core level photoemission of chemisorbed molecules: ethylene and ethylidyne on Rh(111). *Chem. Phys. Lett.* **269,** 371–377 (1997).

14. Blackman, G. S. . *et al.* LEED and HREELS studies of the coadsorbed CO + ethylidyne and NO + ethylidyne systems on the Rh(111) crystal surface. *Surf. Sci.* **207,** 66–88 (1988).

15. Weststrate, C. J. & Niemantsverdriet, J. W. Understanding FTS selectivity: the crucial role of surface hydrogen. *Faraday Discuss.* **197,** 101–116 (2016).

16. Weststrate, C. J., Ciobîcă, I. M., Saib, A. M., Moodley, D. J. & Niemantsverdriet, J. W. Fundamental issues on practical Fischer–Tropsch catalysts: How surface science can help. *Catal. Today* **228,** 106–112 (2014).

17. Liu, J. X., Su, H. Y. & Li, W. X. Structure sensitivity of CO methanation on Co (0001), (101-2) and (112-0) surfaces: Density functional theory calculations. *Catal. Today* **215,** 36–42 (2013).

18. Saib, A. M. *et al.* Fundamental science of cobalt catalyst oxidation and reduction applied to the development of a commercial fischer-tropsch regeneration process. *Ind. Eng. Chem. Res.* **53,** 1816–1824 (2014).

19. Chiang, C.-M. & Bent, B. E. Methyl radical adsorption on Cu(111): bonding, reactivity, and the effect of coadsorbed iodine. *Surf. Sci.* **279,** 79–88 (1992).

20. Cheng, J. *et al.* A DFT study of the chain growth probability in Fischer-Tropsch synthesis. *J. Catal.* **257,** 221–228 (2008).

21. Barneveld, W. A. A. van & Ponec, V. Reactions of CHxCl4-x with Hydrogen : Relation to the Synthesis of Hydrocarbons. *J. Catal.* **387,** 382–387 (1984).

22. Den Breejen, J. P. *et al.* On the origin of the cobalt particle size effects in Fischer-Tropsch catalysis. *J. Am. Chem. Soc.* **131,** 7197–7203 (2009).

23. Kizilkaya, A. C., Niemantsverdriet, J. W. & Weststrate, C. J. Oxygen Adsorption and Water Formation on Co(0001). *J. Phys. Chem. C* **120,** 4833–4842 (2016).

24. Caglar, B., J.W., N. & Weststrate, C. J. Modeling the surface chemistry of biomass model compounds on oxygen-covered Rh(100). *Phys. Chem. Chem. Phys.* **18,** 23888–23903 (2016).

25. Zhuo, M., Borgna, A. & Saeys, M. Effect of the CO coverage on the Fischer-Tropsch synthesis mechanism on cobalt catalysts. *J. Catal.* **297,** 217–226 (2013).
